# Supplementary material for: A competence-regulated toxin-antitoxin system in Haemophilus influenzae
Source: PLoS One. 2020 Jan 13;15(1):e0217255. doi: 10.1371/journal.pone.0217255 (PMC6957337; doi:10.1371/journal.pone.0217255)
Supplement: S1 File — RNAseq analysis methods, Tables A-E, and Figures A-L. (DOCX) [file pone.0217255.s001.docx]

**Contents of this Supplementary Information file:**

- **RNAseq analysis methods**
- **Supplementary Table A: Bacterial strains used in this work:**
- **Supplementary Table B: Metadata for *H. influenzae* strains**
- **Supplementary Table C: *toxTA* genotypes of strains with known competence phenotypes**
- **Supplementary Table D: *H. influenzae* genes whose RNA-seq expression was significantly altered by competence induction**
- **Supplementary Table E: *H. influenzae* genes whose RNA-seq expression was significantly altered in ∆*toxA* relative to wildtype, to ∆*toxT* and to ∆∆*toxTA***
- **Supplementary Figures A-L (list of titles at end of manuscript)**

**Analysis of RNAseq data for wildtype cells:** To identify all genes whose wildtype expression differed between sBHI and MIV, DESeq2 was used to compare RNA-seq expression values in wild-type RNA samples before and after competence induction (T=0 and T=30) [1]. Of the 1747 genes examined, 325 had significantly different expression (adjusted p-value < 0.05 after performing a Wald test and Benjamini-Hoschberg correction in DESeq2). To focus on genes with large changes in expression, we imposed an additional requirement that expression be changed by at least 4-fold, the same threshold used in the previous microarray study [2]. This higher stringency gave 123 genes significantly decreased and 71 significantly increased, for a total of 194/1747 or 11% of all genes tested (listed in Supp. Table 4). Of these, 130 were among the 192 genes previously identified as differentially expressed in the microarray study.

Many of these changes are likely due to the absence of many essential nutrients from MIV medium. The significant induction of 9 of the 10 genes regulated by PurR is expected, since MIV lacks nucleotide precursors; the tenth PurR-regulated gene, HI1616, was also strongly induced in all replicates. MIV also lacks tryptophan, and all 11 Trp-regulon genes regulated by TrpR were significantly induced. The nutritional downshift is also likely to be responsible for the induction of many permeases and transporters, and for the significant downregulation of 39 of the 51 genes encoding ribosomal proteins (*rps* and *rpl* genes). The other 22 ribosomal protein genes were also downregulated, the majority with adjusted p-values < 0.05. Expression of 16S and 23S rRNAs was not measured since these molecules had been depleted from the samples during sequencing library preparation, but expression of the tRNA^Ala^, tRNA^Leu^, and tRNA^Gly^ genes encoded within the six rRNA operons was also significantly reduced.

**Analysis of RNAseq data for competence-regulation mutants:**  To clarify the roles of competence-regulating genes in the observed MIV-induced gene expression changes, RNA-seq coverage values from *sxy* and *crp* knockout strains were compared to values for KW20 sampled at the same four sBHI and MIV timepoints. To evaluate changes at all timepoints simultaneously, a likelihood ratio test was performed using DESeq2 to identify genes that behaved differently between strains. Significant genes were flagged after adjusting for multiple hypothesis testing. Sxy is the competence-specific regulator, and deleting it significantly reduced expression of 24 of the known competence genes [2,3]. Two other previously identified competence genes, HI0250 (*ssb*) and HI1631, also had reduced expression, but these did not meet the significance cutoff. *ssb* is an essential gene [3]; its high baseline expression increased 40% on transfer to MIV and returned to normal in the *sxy* knockout. Expression of HI1631 was reduced 6-fold by the *sxy* knockout, but high variability in KW20 expression led to an insignificant adjusted P-value.

**ToxT and ToxA effects on genes not directly associated with competence:**

Supplementary Table 5 lists, for each timepoint, the genes whose expression in the ∆*toxA* mutant differed significantly from expression in all three strains with normal competence (wildtype, ∆*toxT* and ∆*toxTA*). The most consistent effect was overexpression of the HI0655-58 operon immediately downstream from *toxTA*. Analysis of sequencing coverage in this region showed this to be caused by read-through transcription from both *toxTA* promoters (CRP-S and P2). The operon includes genes encoding shikimate dehydrogenase, an ABC transporter, and a hypothetical protein with putative topoisomerase I domains. Expression of genes in this operon increased about 1.2-1.5-fold in MIV in wildtype cells and in other mutants with normal competence, so their higher expression in ∆*toxA* is unlikely to be responsible for this strain’s competence defect.

Several other genes showed consistent changes relative to most or all competent strains, but these only sometimes reached statistical significance. HI0231 (*deaD*) encodes a DEADbox helicase involved in ribosome assembly and mRNA decay [4]; its expression in control strains fell rapidly on transfer to MIV, but levels in ∆*toxA* were about 50% higher at all time points. HI0235 contains an ArfA ribosome-rescue domain [5]; its expression was up 3-5-fold at t=30 and 2-3-fold at t=100 in the ∆*toxT* and ∆*toxTA* comparisons, but not in the KW20 comparison. HI0362 encodes a CRP-regulated iron-transport protein that normally increases in MIV but did not increase in *toxA* deletion mutants. HI0504 (*rbsB*, a ribose transporter component) is normally induced 20-fold more in MIV than other genes in its operon, but this increase was smaller (only 10-fold) in ∆*toxA*. Expression of HI0595 (*arcC*, carbamate kinase) normally falls 2-3-fold immediately after transfer to MIV, but the fall was greater in ∆*toxA*. 28 other genes were significantly changed only at t=100, but their expression patterns and predicted functions were diverse and did not suggest an explanation for ∆*toxA*’s lack of competence.

**Supplementary Table A**

**Bacterial strains used in this work:**

| **Species** | **Genotype** | **Notes** |
| --- | --- | --- |
| *E. coli* DH5α | F80*lacZ* ∆(*lacIZYA-argF*) *endA1* | Taylor et al. 1993 [6] |
| *H. influenzae* KW20 Rd | wildtype | Fleischman et al. 1995 [7] |
| *H. influenzae* MAP7 | Nov^R^ Nal^R^ Str^R^ Kan^R^ Spc^R^ Vio^R^ | Poje and Redfield 2002 [8] |
| *H. influenzae* | ∆HI0659 | Sinha et al. 2012 [9] |
| *H. influenzae* | ∆HI0660::spc | Sinha et al. 2012 [9] |
| *H. influenzae* | ∆HI0659-0660::spc | This work |
| *H. influenzae* | ∆crp | Chandler et al. 1990 [10] |
| *H. influenzae* | ∆sxy | Williams et al. 1994 [11] |
| *A. pleuropneumoniae* (HS143) | Wildtype | Blackall *et al.* 2002 [12] |
| *A. pleuropneumoniae* | ∆APL_1357::spec | This work |
| *A. pleuropneumoniae* | ∆APL_1358::spec | This work |
| *A. pleuropneumoniae* | ∆APL_1357-1358::spec | This work |

**Supplementary Table B**

**Metadata for *H. influenzae* strains**

| **strain** | **toxA** | **toxT** | **toxTA deletion** | **collection** | **collection location** | **Date** | **patient age** | **health state** | **body site** | **disease** | **isolation source** |
| --- | --- | --- | --- | --- | --- | --- | --- | --- | --- | --- | --- |
| 10810 | present | present | no deletion | ncbi | UK | unkn | unkn | sick | unkn | meningitis | unkn |
| 1104 | present | present | removed 178bp of toxT, both promoters intact | ncbi | Spain: Barcelona, Catalonia | 1998 | 56 yr | sick | lung | bronchiectasis | sputum |
| 1209 | present | present | removed 178bp of toxT, both promoters intact | ncbi | Finland | 1994 | child | sick | ear | OM | MEF |
| 22.1-21 | present | present | removed both toxTA promoters and toxT start codon | Gilsdorf | USA: Ann Arbor, MI | 1998 | child | healthy | np | carriage | np swab |
| 22.4-21 | present | present | no deletion | Gilsdorf | USA: Ann Arbor, MI | 1998 | child | healthy | np | carriage | np swab |
| 2842STDY5882018 | present | absent | removed both toxTA promoters and toxT start codon | ncbi | The Netherlands | unkn | child | sick | csf | meningitis | csf |
| 2842STDY5882019 | present | absent | removed both toxTA promoters and toxT start codon | ncbi | The Netherlands | unkn | child | sick | bld | meningitis | bld |
| 2842STDY5882021 | present | present | removed 178bp of toxT, both promoters intact | ncbi | The Netherlands | unkn | child | sick | csf | meningitis | csf |
| 2842STDY5882022 | present | present | removed 178bp of toxT, both promoters intact | ncbi | The Netherlands | unkn | child | sick | bld | meningitis | bld |
| 2842STDY5882032 | present | present | removed 178bp of toxT, both promoters intact | ncbi | The Netherlands | unkn | child | sick | csf | meningitis | csf |
| 2842STDY5882033 | present | present | removed 178bp of toxT, both promoters intact | ncbi | The Netherlands | unkn | child | sick | bld | meningitis | bld |
| 2842STDY5882034 | present | present | removed 178bp of toxT, both promoters intact | ncbi | The Netherlands | unkn | child | sick | csf | meningitis | csf |
| 2842STDY5882035 | present | present | removed 178bp of toxT, both promoters intact | ncbi | The Netherlands | unkn | child | sick | bld | meningitis | bld |
| 2842STDY5882036 | present | present | removed 178bp of toxT, both promoters intact | ncbi | The Netherlands | unkn | child | sick | csf | meningitis | csf |
| 2842STDY5882037 | present | present | removed 178bp of toxT, both promoters intact | ncbi | The Netherlands | unkn | child | sick | bld | meningitis | bld |
| 2842STDY5882040 | present | absent | removed both toxTA promoters and toxT start codon | ncbi | The Netherlands | unkn | child | sick | csf | meningitis | csf |
| 2842STDY5882041 | present | absent | removed both toxTA promoters and toxT start codon | ncbi | The Netherlands | unkn | child | sick | bld | meningitis | bld |
| 2842STDY5882045 | present | present | removed 178bp of toxT, both promoters intact | ncbi | The Netherlands | unkn | child | sick | csf | meningitis | csf |
| 2842STDY5882046 | present | present | removed 178bp of toxT, both promoters intact | ncbi | The Netherlands | unkn | child | sick | bld | meningitis | bld |
| 2842STDY5882047 | present | absent | removed both toxTA promoters and toxT start codon | ncbi | The Netherlands | unkn | child | sick | csf | meningitis | csf |
| 2842STDY5882048 | present | absent | removed both toxTA promoters and toxT start codon | ncbi | The Netherlands | unkn | child | sick | bld | meningitis | bld |
| 2842STDY5882049 | present | present | no deletion | ncbi | The Netherlands | unkn | child | sick | csf | meningitis | csf |
| 2842STDY5882050 | present | present | no deletion | ncbi | The Netherlands | unkn | child | sick | bld | meningitis | bld |
| 2842STDY5882051 | present | present | removed 178bp of toxT, both promoters intact | ncbi | The Netherlands | unkn | child | sick | csf | meningitis | csf |
| 2842STDY5882052 | present | present | removed 178bp of toxT, both promoters intact | ncbi | The Netherlands | unkn | child | sick | bld | meningitis | bld |
| 2842STDY5882055 | present | present | removed 178bp of toxT, both promoters intact | ncbi | The Netherlands | unkn | child | sick | csf | meningitis | csf |
| 2842STDY5882056 | present | present | removed 178bp of toxT, both promoters intact | ncbi | The Netherlands | unkn | child | sick | bld | meningitis | bld |
| 2842STDY5882057 | present | absent | removed both toxTA promoters and toxT start codon | ncbi | The Netherlands | unkn | child | sick | csf | meningitis | csf |
| 2842STDY5882058 | present | absent | removed both toxTA promoters and toxT start codon | ncbi | The Netherlands | unkn | child | sick | bld | meningitis | bld |
| 2842STDY5882059 | present | absent | removed both toxTA promoters and toxT start codon | ncbi | The Netherlands | unkn | child | sick | csf | meningitis | csf |
| 2842STDY5882060 | present | absent | removed both toxTA promoters and toxT start codon | ncbi | The Netherlands | unkn | child | sick | bld | meningitis | bld |
| 2842STDY5882061 | present | present | removed 178bp of toxT, both promoters intact | ncbi | The Netherlands | unkn | child | sick | csf | meningitis | csf |
| 2842STDY5882062 | present | present | removed 178bp of toxT, both promoters intact | ncbi | The Netherlands | unkn | child | sick | bld | meningitis | bld |
| 2842STDY5882064 | absent | absent | toxTA absent | ncbi | The Netherlands | unkn | child | sick | csf | meningitis | csf |
| 2842STDY5882065 | absent | absent | toxTA absent | ncbi | The Netherlands | unkn | child | sick | bld | meningitis | bld |
| 2842STDY5882075 | present | present | no deletion | ncbi | The Netherlands | unkn | child | sick | csf | meningitis | csf |
| 2842STDY5882076 | present | present | no deletion | ncbi | The Netherlands | unkn | child | sick | bld | meningitis | bld |
| 2842STDY5882078 | present | present | removed 178bp of toxT, both promoters intact | ncbi | The Netherlands | unkn | child | sick | csf | meningitis | csf |
| 2842STDY5882079 | present | present | removed 178bp of toxT, both promoters intact | ncbi | The Netherlands | unkn | child | sick | bld | meningitis | bld |
| 2842STDY5882080 | present | present | removed 178bp of toxT, both promoters intact | ncbi | The Netherlands | unkn | child | sick | csf | meningitis | csf |
| 2842STDY5882081 | present | present | removed 178bp of toxT, both promoters intact | ncbi | The Netherlands | unkn | child | sick | bld | meningitis | bld |
| 2842STDY5882085 | present | present | removed 178bp of toxT, both promoters intact | ncbi | The Netherlands | unkn | child | sick | csf | meningitis | csf |
| 2842STDY5882086 | present | present | removed 178bp of toxT, both promoters intact | ncbi | The Netherlands | unkn | child | sick | bld | meningitis | bld |
| 2842STDY5882087 | present | present | removed 178bp of toxT, both promoters intact | ncbi | The Netherlands | unkn | child | sick | csf | meningitis | csf |
| 2842STDY5882088 | present | present | removed 178bp of toxT, both promoters intact | ncbi | The Netherlands | unkn | child | sick | bld | meningitis | bld |
| 3655 | present | absent | removed both toxTA promoters and toxT start codon | ncbi | USA: St. Louis, MO | unkn | child | sick | ear | OM | ear fluid |
| 40 HINF | absent | absent | toxTA absent | ncbi | USA: Seattle, WA | 2012-2013 | 39.3 yr | sick | lung | COPD | sputum |
| 411 | present | present | removed 178bp of toxT, both promoters intact | ncbi | Spain: Barcelona, Catalonia | 1997 | 56 yr | sick | lung | bronchiectasis | sputum |
| 477 | present | present | removed 178bp of toxT, both promoters intact | ncbi | Finland | 1994 | child | sick | ear | OM | ear fluid |
| 536 HINF | present | present | removed 178bp of toxT, both promoters intact | ncbi | USA: Seattle, WA | 2012-2013 | 49.5 yr | sick | lung | bronchiectasis | bal |
| 552 HINF | present | present | removed 178bp of toxT, both promoters intact | ncbi | USA: Seattle, WA | 2012-2013 | 49.5 yr | sick | lung | bronchiectasis | bal |
| 584 | present | present | removed 178bp of toxT, both promoters intact | ncbi | Spain: Barcelona, Catalonia | 1997 | 56 yr | sick | lung | bronchiectasis | sputum |
| 60294N1 | present | present | removed 178bp of toxT, both promoters intact | ncbi | Australia: North Territory | 2008 | 4.2 yr | sick | np | bronchiectasis | np swab |
| 6P18H1 | present | present | removed 178bp of toxT, both promoters intact | ncbi | USA: Buffalo, NY | unkn | 72 yr | sick | lung | COPD | sputum |
| 723 | absent | absent | toxTA absent | ncbi | Finland | 1994 | child | sick | ear | OM | ear fluid |
| 7P49H1 | present | present | removed 178bp of toxT, both promoters intact | ncbi | USA: Buffalo, NY | unkn | 65 yr | sick | lung | COPD | sputum |
| 86-028NP | present | present | removed 178bp of toxT, both promoters intact | ncbi | USA: Columbus, OH | 1987 | child | sick | np | OM | np swab |
| CGSHiCZ412602 | present | absent | removed both toxTA promoters and toxT start codon | ncbi | Czech Republic | unkn | child | sick | ear | OM | MEF |
| DC7331102 | absent | absent | toxTA absent | pnas | UK | 1997 | unkn | sick | unkn | meningitis | unkn |
| F3031 | absent | absent | toxTA absent | ncbi | Brazil: Serrana | 1984-1986 | unkn | sick | bld | Brazilian purpuric fever | blood |
| F3047 | present | present | no deletion | ncbi | Brazil: Guariba | 1984-1986 | unkn | sick | conjunctiva | conjunctivitis | eye swab |
| HI1373 | present | present | removed 178bp of toxT, both promoters intact | ncbi | USA: Ohio | unkn | unkn | sick | ear | OM | ear fluid |
| HI1374 | present | absent | removed both toxTA promoters and toxT start codon | ncbi | USA: Ohio | unkn | unkn | sick | csf | meningitis | csf |
| HI1388 | present | absent | removed both toxTA promoters and toxT start codon | ncbi | USA: Missouri | unkn | unkn | sick | ear | OM | ear fluid |
| HI1394 | present | absent | removed both toxTA promoters and toxT start codon | ncbi | USA: Texas | unkn | unkn | sick | ear | OM | ear fluid |
| HI1408 | present | present | removed 178bp of toxT, both promoters intact | ncbi | Canada | unkn | unkn | sick | csf | meningitis | csf |
| HI1417 | present | absent | removed both toxTA promoters and toxT start codon | ncbi | USA: Texas | unkn | unkn | sick | ear | OM | ear fluid |
| HI1426 | present | absent | removed both toxTA promoters and toxT start codon | ncbi | USA: Minnesota | unkn | unkn | sick | csf | meningitis | csf |
| HI1722 | present | absent | removed both toxTA promoters and toxT start codon | ncbi | USA: Columbus, OH | unkn | child | sick | ear | OM | ear fluid |
| HI1974 | absent | absent | toxTA absent | ncbi | USA: Oklahoma City, OK | 2004 | 79 yr | sick | bld | invasive | bld |
| HI1980 | present | absent | removed both toxTA promoters and toxT start codon | ncbi | USA: Oklahoma City, OK | 2004 | 37 yr | sick | bld | invasive | bld |
| HI1988 | present | present | removed 178bp of toxT, both promoters intact | ncbi | USA: Oklahoma City, OK | unkn | 1 day | sick | bld | invasive | bld |
| HI2004 | present | present | removed 178bp of toxT, both promoters intact | ncbi | USA: Oklahoma City, OK | 2003 | 24 yr | sick | bld | bacteremia | bld |
| HI2007 | present | present | removed 178bp of toxT, both promoters intact | ncbi | USA: Oklahoma City, OK | 2004 | 3 weeks | sick | bld | bacteremia | bld |
| HI2116 | present | present | removed 178bp of toxT, both promoters intact | ncbi | USA: Arkansas | unkn | 3 yr | sick | bld | bacteremia | bld |
| HI2192 | present | absent | removed both toxTA promoters and toxT start codon | ncbi | USA: Oklahoma | 2008 | child | sick | ear | OM | ear fluid |
| HI2428 | present | present | no deletion | ncbi | USA: Oklahoma City, OK | 1905 | unkn | sick | synovial joint | septic arthritis | synovial fluid |
| Hi1008 | present | present | no deletion | pnas | Finland | 1994-1995 | child | sick | ear | OM | ear fluid |
| Hi11 | present | present | removed 178bp of toxT, both promoters intact | pnas | Finland | unkn | child | healthy | np | carriage | np swab |
| Hi1124 | present | present | removed 178bp of toxT, both promoters intact | pnas | Finland | 1994-1995 | child | sick | ear | OM | ear fluid |
| Hi1158 | absent | absent | toxTA absent | pnas | Finland | 1994-1995 | child | sick | ear | OM | ear fluid |
| Hi1180 | present | absent | removed both toxTA promoters and toxT start codon | pnas | Finland | 1994-1995 | child | sick | ear | OM | ear fluid |
| Hi1200 | present | present | removed 178bp of toxT, both promoters intact | pnas | Finland | 1994-1995 | child | sick | ear | OM | ear fluid |
| Hi1207 | present | present | removed 178bp of toxT, both promoters intact | pnas | Finland | 1994-1995 | child | sick | ear | OM | ear fluid |
| Hi1231 | present | present | removed 178bp of toxT, both promoters intact | pnas | Finland | 1994-1995 | child | sick | ear | OM | ear fluid |
| Hi1233 | present | present | removed 178bp of toxT, both promoters intact | pnas | Finland | 1994-1995 | child | sick | ear | OM | ear fluid |
| Hi1247 | present | present | removed 178bp of toxT, both promoters intact | pnas | Finland | 1994-1995 | child | sick | ear | OM | ear fluid |
| Hi1268 | present | present | removed 178bp of toxT, both promoters intact | pnas | Finland | 1994-1995 | child | sick | ear | OM | ear fluid |
| Hi1363 | present | present | removed 178bp of toxT, both promoters intact | pnas | Finland | unkn | child | sick | ear | OM | ear fluid |
| Hi1513 | present | absent | removed both toxTA promoters and toxT start codon | pnas | Spain: Barcelona | 2008 | adult | sick | lung | COPD | sputum |
| Hi1549 | present | present | removed 178bp of toxT, both promoters intact | pnas | Spain: Barcelona | 2008 | adult | sick | lung | COPD | transthoracic needle aspiration fluid |
| Hi1553 | present | absent | removed both toxTA promoters and toxT start codon | pnas | Spain: Barcelona | 2008 | adult | sick | lung | COPD | sputum |
| Hi1556 | absent | absent | toxTA absent | pnas | Spain: Barcelona | 2008 | adult | sick | lung | COPD | sputum |
| Hi1557 | present | absent | removed both toxTA promoters and toxT start codon | pnas | Spain: Barcelona | 2008 | adult | sick | lung | COPD | sputum |
| Hi1558 | present | present | removed 178bp of toxT, both promoters intact | pnas | Spain: Barcelona | 2008 | unkn | sick | lung | bronchiectasis | sputum |
| Hi1559 | absent | absent | toxTA absent | pnas | Spain: Barcelona | 2008 | unkn | sick | lung | pneumonia | bal |
| Hi1560 | present | present | removed 178bp of toxT, both promoters intact | pnas | Spain: Barcelona | 2008 | adult | sick | lung | COPD | sputum |
| Hi1566 | absent | absent | toxTA absent | pnas | Spain: Barcelona | 2008 | adult | sick | lung | COPD | sputum |
| Hi1568 | present | present | removed 178bp of toxT, both promoters intact | pnas | Spain: Barcelona | 2008 | adult | sick | lung | COPD | sputum |
| Hi16 | present | present | removed 178bp of toxT, both promoters intact | pnas | Finland | unkn | child | healthy | np | carriage | np swab |
| Hi1606 | present | present | no deletion | pnas | Spain: Mallorca | 2008 | unkn | sick | lung | bronchiectasis | sputum |
| Hi1607 | absent | absent | toxTA absent | pnas | Spain: Mallorca | 2008 | adult | sick | lung | COPD | sputum |
| Hi1619 | present | present | removed 178bp of toxT, both promoters intact | pnas | Spain: Mallorca | 2008 | unkn | sick | np | CF | np swab |
| Hi162 | present | absent | removed both toxTA promoters and toxT start codon | pnas | Finland | 1994-1995 | child | sick | ear | OM | ear fluid |
| Hi1621 | present | absent | removed both toxTA promoters and toxT start codon | pnas | Spain: Mallorca | 2008 | adult | sick | bronchial tube | COPD | bronchial aspirate |
| Hi1622 | present | present | removed 178bp of toxT, both promoters intact | pnas | Spain: Mallorca | 2008 | unkn | sick | lung | bronchiolitis | sputum |
| Hi1623 | present | absent | removed both toxTA promoters and toxT start codon | pnas | Spain: Mallorca | 2008 | adult | sick | lung | COPD | sputum |
| Hi1630 | present | present | removed 178bp of toxT, both promoters intact | pnas | Spain: Mallorca | 2008 | adult | sick | lung | acute tracheobronchiolitis | sputum |
| Hi167 | present | absent | removed both toxTA promoters and toxT start codon | pnas | Finland | unkn | child | sick | ear | OM | ear fluid |
| Hi17 | present | present | removed 178bp of toxT, both promoters intact | pnas | Finland | unkn | child | healthy | np | carriage | np swab |
| Hi176 | present | present | removed 178bp of toxT, both promoters intact | pnas | Finland | 1994 | child | sick | ear | OM | ear fluid |
| Hi177 | present | present | removed 178bp of toxT, both promoters intact | pnas | Finland | unkn | child | healthy | np | carriage | np swab |
| Hi199 | present | present | removed 178bp of toxT, both promoters intact | pnas | Finland | unkn | child | sick | ear | OM | ear fluid |
| Hi206 | present | present | removed 178bp of toxT, both promoters intact | pnas | Finland | unkn | child | sick | ear | OM | ear fluid |
| Hi24 | present | present | removed 178bp of toxT, both promoters intact | pnas | Finland | 1994 | child | healthy | np | carriage | np swab |
| Hi264 | present | present | removed 178bp of toxT, both promoters intact | pnas | Finland | unkn | child | healthy | np | carriage | np swab |
| Hi285 | present | present | removed 178bp of toxT, both promoters intact | pnas | Finland | 1994 | child | sick | ear | OM | ear fluid |
| Hi322 | present | absent | removed both toxTA promoters and toxT start codon | ncbi | Italy: Pisa | 39896 | 3 yr | sick | csf | meningitis | csf |
| Hi345 | present | present | removed 178bp of toxT, both promoters intact | ncbi | Italy: Rimini | 39960 | 73 yr | sick | bld | invasive | bld |
| Hi359 | absent | absent | toxTA absent | ncbi | Italy: Desensano | 40277 | 74 yr | sick | bld | invasive | bld |
| Hi361 | present | present | removed 178bp of toxT, both promoters intact | ncbi | Italy: Milano | 40179 | 72 yr | sick | bld | invasive | bld |
| Hi378 | absent | absent | toxTA absent | ncbi | Italy: Trento | 40493 | 70 yr | sick | csf | meningitis | csf |
| Hi381 | present | present | removed 178bp of toxT, both promoters intact | ncbi | Italy: Brescia | 40546 | 79 yr | sick | csf | meningitis | csf |
| Hi394 | present | present | removed 178bp of toxT, both promoters intact | ncbi | Italy: Brescia | 40590 | 43 yr | sick | bld | invasive | bld |
| Hi398 | present | absent | removed both toxTA promoters and toxT start codon | pnas | Spain: Mallorca | 1995 | adult | sick | lung | COPD | sputum |
| Hi403 | present | present | removed 178bp of toxT, both promoters intact | ncbi | Italy: Ivria | 2011 | 65 yr | sick | csf | meningitis | csf |
| Hi432 | present | absent | removed both toxTA promoters and toxT start codon | pnas | Finland | 1994-1995 | child | sick | ear | OM | ear fluid |
| Hi443 | present | absent | removed both toxTA promoters and toxT start codon | pnas | Finland | unkn | child | healthy | np | carriage | np swab |
| Hi492 | absent | absent | toxTA absent | pnas | Finland | unkn | child | healthy | np | carriage | np swab |
| Hi525 | present | absent | removed both toxTA promoters and toxT start codon | pnas | Finland | unkn | child | healthy | np | carriage | np swab |
| Hi609 | present | present | no deletion | pnas | Finland | unkn | child | healthy | np | carriage | np swab |
| Hi639 | present | present | removed 178bp of toxT, both promoters intact | pnas | Finland | 1994-1995 | child | sick | ear | OM | ear fluid |
| Hi658 | present | present | removed 178bp of toxT, both promoters intact | pnas | Finland | 1994-1995 | child | sick | ear | OM | ear fluid |
| Hi667 | present | present | removed 178bp of toxT, both promoters intact | pnas | Finland | 1994-1995 | child | sick | ear | OM | ear fluid |
| Hi709 | absent | absent | toxTA absent | pnas | Finland | unkn | child | healthy | np | carriage | np swab |
| Hi740 | present | present | removed 178bp of toxT, both promoters intact | pnas | Finland | 1994-1995 | child | sick | ear | OM | ear fluid |
| Hi787 | absent | absent | toxTA absent | pnas | Finland | unkn | child | healthy | np | carriage | np swab |
| Hi794 | present | present | no deletion | pnas | Finland | unkn | child | healthy | np | carriage | np swab |
| Hi805 | present | present | removed 178bp of toxT, both promoters intact | pnas | Finland | 1994-1995 | child | healthy | np | carriage | np swab |
| Hi88 | present | absent | removed both toxTA promoters and toxT start codon | pnas | Finland | unkn | child | healthy | np | carriage | np swab |
| Hi968 | present | present | removed 178bp of toxT, both promoters intact | pnas | Finland | 1994-1995 | child | healthy | np | carriage | np swab |
| Hi973 | present | present | removed 178bp of toxT, both promoters intact | pnas | Finland | 1994-1995 | child | sick | ear | OM | ear fluid |
| Hi981 | present | absent | removed both toxTA promoters and toxT start codon | pnas | Finland | 1994-1995 | child | sick | ear | OM | ear fluid |
| HiR3021 | present | present | removed 178bp of toxT, both promoters intact | pnas | The Netherlands: Amsterdam | 1995 | child | healthy | np | carriage | np swab |
| KR494 | present | present | no deletion | ncbi | Sweden: Malmö | 2010 | 70 yr | sick | unkn | necrotizing myositis, sepsis | bld |
| MiHi270 | present | present | removed 178bp of toxT, both promoters intact | ncbi | Italy: Milano | 2012 | 6 yr | healthy | np | carriage | np swab |
| NCTC8143 | present | absent | removed both toxTA promoters and toxT start codon | ncbi | UK | 1950 | unkn | unkn | unkn | unkn | unkn |
| NT127 | present | present | no deletion | ncbi | USA: Boston, MA | unkn | 6 months | sick | csf | meningitis | csf |
| PittEE | present | present | no deletion | Pittsburgh | USA: Pittsburgh, PA | 2000 | child | sick | ear | OM | ear fluid |
| PittGG | present | present | no deletion | Pittsburgh | USA: Pittsburgh, PA | 2000 | child | sick | ear | otorrhea | ear fluid |
| RM600672 | present | present | no deletion | pnas | unkn | unkn | unkn | unkn | unkn | unkn | unkn |
| RM601173 | absent | absent | toxTA absent | pnas | UK | 1984 | unkn | sick | unkn | meningitis | unkn |
| RM601974 | absent | absent | toxTA absent | pnas | UK | 1984 | unkn | sick | unkn | meningitis | unkn |
| RM603375 | present | absent | removed both toxTA promoters and toxT start codon | pnas | UK | 1984 | unkn | sick | fallopian tube | pus hydrosalpinx | pus |
| RM605177 | present | present | removed 178bp of toxT, both promoters intact | pnas | UK | 1985 | unkn | sick | csf | meningitis | csf |
| RM701878 | present | present | removed 178bp of toxT, both promoters intact | pnas | Ghana | 1983 | unkn | sick | csf | meningitis | csf |
| RM702879 | present | absent | removed both toxTA promoters and toxT start codon | pnas | Papua New Guinea | 1980s | unkn | sick | bld | invasive | bld |
| RM702980 | present | present | removed 178bp of toxT, both promoters intact | pnas | Papua New Guinea | 1980s | unkn | sick | bld | invasive | bld |
| RM706883 | present | present | no deletion | pnas | Papua New Guinea | unkn | unkn | sick | lung | pneumonia | sputum |
| RM712284 | present | present | removed 178bp of toxT, both promoters intact | pnas | Australia | <1984 | unkn | sick | unkn | meningitis | unkn |
| RM730885 | present | absent | removed both toxTA promoters and toxT start codon | pnas | South Korea | 1984 | unkn | healthy | np | carriage | np swab |
| RM730986 | present | present | removed 178bp of toxT, both promoters intact | pnas | South Korea | 1984 | unkn | healthy | np | carriage | np swab |
| RM734787 | present | present | removed 178bp of toxT, both promoters intact | pnas | USA | 1985 | unkn | sick | lung | unkn | sputum |
| RM744888 | present | present | removed 178bp of toxT, both promoters intact | pnas | Iceland | 1978 | unkn | sick | bld | invasive | bld |
| RM745989 | present | present | no deletion | pnas | Iceland | 1984 | unkn | sick | csf | meningitis | csf |
| RM746590 | present | present | no deletion | pnas | Iceland | 1985 | unkn | sick | csf | meningitis | csf |
| RM747791 | present | present | removed 178bp of toxT, both promoters intact | pnas | Iceland | 1986 | unkn | unkn | unkn | unkn | unkn |
| RM749092 | present | absent | removed both toxTA promoters and toxT start codon | pnas | South Africa | 1980s | unkn | sick | csf | meningitis | csf |
| RM761793 | present | present | no deletion | pnas | Malaysia | 1970s | unkn | sick | csf | meningitis | csf |
| RM763794 | present | present | no deletion | pnas | China | 1971 | unkn | sick | lung | unkn | sputum |
| RM787695 | present | present | removed 178bp of toxT, both promoters intact | pnas | unkn | unkn | unkn | unkn | unkn | unkn | unkn |
| RMHi93 | present | present | removed 178bp of toxT, both promoters intact | ncbi | Italy: Rome | 2012 | 9 months | healthy | oropharyngeal | carriage | oropharyngeal swab |
| Rd KW20 | present | present | no deletion | ncbi | USA: New York, NY | 1943-44 | child | unkn | nasopharynx | unkn | np swab |
| RdAW | present | present | no deletion | ncbi | USA: Ann Arbor, MI | 2002 | unkn | unkn | nasopharynx | unkn | unkn |
| 2019 | present | present | removed 178bp of toxT, both promoters intact | ncbi | USA: Buffalo, NY | unkn | adult | sick | lung | chronic bronchitis | sputum |
| C486 | present | absent | removed both toxTA promoters and toxT start codon | ncbi | USA: Seattle, WA | 1978 | child | sick | ear | OM | ear fluid |
| Hi375 | present | absent | removed both toxTA promoters and toxT start codon | ncbi | Finland | 1994 | child | sick | ear | OM | ear fluid |
| PittAA | present | present | removed 178bp of toxT, both promoters intact | Pittsburgh | USA: Pittsburgh, PA | 2000 | child | sick | ear | OM | ear fluid |
| PittII | present | absent | removed both toxTA promoters and toxT start codon | ncbi | USA: Pittsburgh, PA | 2000 | child | sick | ear | otorrhea | ear fluid |
| R2846 | present | absent | removed both toxTA promoters and toxT start codon | ncbi | USA: St. Louis, MO | unkn | child | sick | ear | OM | ear fluid |
| R2866 | present | present | removed 178bp of toxT, both promoters intact | ncbi | USA: Seattle, WA | 1994 | 30 months | sick | bld | meningitis | bld |

**Supplementary Table C**

**Phenotypes of *H. influenzae* genes with known *toxTA* genotypes**

**(Phenotype data from Maughan *et al*. 2010)**

| **strain** | **toxTA deletion** | **DNA uptake** | **Transformation** | **Doubling time (min)** | **Final OD600** | **cfu/ml** |
| --- | --- | --- | --- | --- | --- | --- |
| 477 | removed 178bp of toxT, both promoters intact | 5.E-02 | none | 45 | 2.3 | 3.E+09 |
| 1209 | removed 178bp of toxT, both promoters intact | 8.E-03 | 7.E-04 | 35 | 1.6 | 5.E+09 |
| 22.1-21 | removed both toxTA promoters and toxT start codon | 7.E-03 | 2.E-06 | 51 | 2.6 | 4.E+08 |
| 22.4-21 | no deletion | 2.E-03 | 1.E-07 | 43 | 3.1 | 1.E+08 |
| 86-028NP | removed 178bp of toxT, both promoters intact | 6.E-03 | 5.E-06 | 58 | 0.1 | 3.E+08 |
| Hi1008 | no deletion | 2.E-02 | 1.E-05 | 50 | 1.7 | 4.E+09 |
| Hi1158 | toxTA absent | 8.E-02 | 1.E-06 | 40 | 2.0 | 3.E+09 |
| Hi1207 | removed 178bp of toxT, both promoters intact | 3.E-02 | 5.E-04 | 53 | 1.6 | 5.E+09 |
| Hi1233 | removed 178bp of toxT, both promoters intact | 8.E-03 | 4.E-04 | 45 | 2.5 | 4.E+09 |
| Hi1247 | removed 178bp of toxT, both promoters intact | 8.E-02 | 1.E-05 | 50 | 2.1 | 6.E+09 |
| Hi375 | removed both toxTA promoters and toxT start codon | 4.E-01 | 1.E-02 | 43 | 1.6 | 6.E+09 |
| Hi432 | removed both toxTA promoters and toxT start codon | 8.E-03 | 2.E-07 | 53 | 1.8 | 7.E+09 |
| PittAA | removed 178bp of toxT, both promoters intact | 1.E-02 | 1.E-02 | 50 | 2.1 | 8.E+09 |
| PittEE | no deletion | 3.E-03 | none | 38 | 0.4 | 3.E+07 |
| PittGG | no deletion | 1.E-02 | 2.E-04 | 40 | 1.8 | 3.E+09 |
| PittII | removed both toxTA promoters and toxT start codon | 8.E-02 | 2.E-06 | 48 | 1.7 | 6.E+07 |
| R2846 | removed both toxTA promoters and toxT start codon | 2.E-03 | 1.E-07 | 50 | 2.9 | 7.E+09 |
| R2866 | removed 178bp of toxT, both promoters intact | 1.E-01 | 1.E-04 | 38 | 2.7 | 7.E+09 |
| Rd KW20 | no deletion | 1.E+00 | 1.E-02 | 45 | 2.3 | 2.E+08 |

**Supplementary Table D**

***H. influenzae* genes whose RNA-seq expression was significantly altered by competence induction**

| **Gene ID** | **Description** | **Gene name** | **logFC** | **Padj** | **Function** |
| --- | --- | --- | --- | --- | --- |
| HI1080 | amino acid ABC transporter substrate-binding protein |  | 4.093 | 0.000 | Other |
| HI1154 | proton glutamate symport protein |  | 3.457 | 0.000 | Other |
| HI1079m | amino acid ABC transporter permease |  | 3.403 | 0.000 | Other |
| HI1078 | amino-acid ABC transporter ATP-binding protein |  | 3.386 | 0.000 | Other |
| HI0779 | 50S ribosomal protein L23 | rplW | 3.352 | 0.000 | Ribos |
| HI0780 | 50S ribosomal protein L2 | rplB | 3.337 | 0.000 | Ribos |
| HI0777 | 50S ribosomal protein L3 | rplC | 3.250 | 0.000 | Ribos |
| HI0776 | 30S ribosomal protein S10 | rpsJ | 3.243 | 0.000 | Ribos |
| HI0778 | 50S ribosomal protein L4 | rplD | 3.235 | 0.000 | Ribos |
| HI0980 | DNA-binding protein Fis | fis | 3.214 | 0.000 | Other |
| HI0781 | 30S ribosomal protein S19 | rpsS | 3.178 | 0.000 | Ribos |
| HI0782 | 50S ribosomal protein L22 | rplV | 3.147 | 0.000 | Ribos |
| HI0231 | ATP-dependent RNA helicase | deaD | 3.024 | 0.000 | Other |
| HI0783 | 30S ribosomal protein S3 | rpsC | 2.984 | 0.000 | Ribos |
| HI0220.6 | tRNA-Glu | tRNA-Glu | 2.972 | 0.000 | tRNA |
| HI0621.5 | tRNA-Glu | tRNA-Glu | 2.940 | 0.000 | tRNA |
| HI1003.4 | tRNA-Glu-1 | tRNA-Glu | 2.912 | 0.000 | tRNA |
| HI1733 | exoribonuclease II | rnb | 2.864 | 0.000 | Other |
| HI0784 | 50S ribosomal protein L16 | rplP | 2.861 | 0.000 | Ribos |
| HI0185 | alcohol dehydrogenase class III | adhC | 2.839 | 0.000 | Other |
| HI0785 | 50S ribosomal protein L29 | rpmC | 2.828 | 0.000 | Ribos |
| HI1282 | hypothetical protein |  | 2.807 | 0.000 | Other |
| HI0682 | ketol-acid reductoisomerase | ilvC | 2.773 | 0.000 | Other |
| HI1177 | arginine transporter permease subunit ArtM | artM | 2.722 | 0.000 | Other |
| HI0786 | 30S ribosomal protein S17 | rpsQ | 2.711 | 0.000 | Ribos |
| HI0974m | pseudogene |  | 2.663 | 0.000 | Other |
| HI0979 | hypothetical protein |  | 2.656 | 0.000 | Other |
| HI0545 | 30S ribosomal protein S18 | rpsR | 2.646 | 0.000 | Ribos |
| HI0641 | 50S ribosomal protein L7/L12 |  | 2.635 | 0.000 | Other |
| HI0640 | 50S ribosomal protein L10 | rplJ | 2.560 | 0.000 | Ribos |
| HI0797 | 50S ribosomal protein L15 | rplO | 2.550 | 0.000 | Ribos |
| HI0796 | 50S ribosomal protein L30 | rpmD | 2.528 | 0.000 | Ribos |
| HI0547 | 30S ribosomal protein S6 | rpsF | 2.526 | 0.000 | Ribos |
| HI0546 | primosomal replication protein N |  | 2.510 | 0.000 | Other |
| HI1180 | arginine transporter ATP-binding protein | artP | 2.505 | 0.000 | Other |
| HI0864 | GTP-binding protein |  | 2.496 | 0.000 | Other |
| HI0544 | 50S ribosomal protein L9 | rplI | 2.492 | 0.000 | Ribos |
| HI0795 | 30S ribosomal protein S5 | rpsE | 2.440 | 0.000 | Ribos |
| HI0184 | esterase |  | 2.430 | 0.000 | Other |
| HI0973 | hypothetical protein |  | 2.402 | 0.000 | Other |
| HI0798 | preprotein translocase subunit SecY | secY | 2.367 | 0.000 | Other |
| HI0853 | heme-binding lipoprotein | dppA | 2.338 | 0.000 | Other |
| HI0157 | 3-oxoacyl-ACP synthase | fabH | 2.308 | 0.000 | Other |
| HI0201 | 50S ribosomal protein L19 | rplS | 2.287 | 0.000 | Ribos |
| HI1060 | lipid-A-disaccharide synthase | lpxB | 2.282 | 0.000 | Other |
| HI0319 | S-adenosyl-L-methionine-dependent methyltransferase | yecO | 2.278 | 0.000 | Other |
| HI0758 | 50S ribosomal protein L31 | rpmE | 2.275 | 0.000 | Ribos |
| HI0202 | tRNA (guanine-N(1)-)-methyltransferase | trmD | 2.275 | 0.000 | Other |
| HI0160 | phosphatidylserine decarboxylase | psd | 2.256 | 0.000 | Other |
| HI0794 | 50S ribosomal protein L18 | rplR | 2.247 | 0.000 | Ribos |
| HI0972 | acetyl-CoA carboxylase biotin carboxylase subunit | accC | 2.228 | 0.000 | Other |
| HI1618 | nickel transport protein | nikO | 2.215 | 0.000 | Other |
| HI1619 | nickel transport protein | nikQ | 2.177 | 0.002 | Other |
| HI1000 | hypothetical protein |  | 2.170 | 0.000 | Other |
| HI0517 | 50S ribosomal protein L11 | rplK | 2.158 | 0.000 | Ribos |
| HI0203 | 16S rRNA-processing protein RimM | rimM | 2.131 | 0.000 | Other |
| HI0810 | Hsp33-like chaperonin | hslO | 2.110 | 0.000 | Other |
| HI1051 | ABC transporter ATP-binding protein |  | 2.110 | 0.000 | Other |
| HI0516 | 50S ribosomal protein L1 | rplA | 2.098 | 0.000 | Ribos |
| HI0793 | 50S ribosomal protein L6 | rplF | 2.097 | 0.000 | Ribos |
| HI1622 | nickel transport protein | nikL | 2.087 | 0.000 | Other |
| HI0803 | 50S ribosomal protein L17 | rplQ | 2.078 | 0.000 | Ribos |
| HI0204 | 30S ribosomal protein S16 | rpsP | 2.072 | 0.000 | Ribos |
| HI1001 | inner membrane protein translocase component YidC | yidC | 2.071 | 0.000 | Other |
| HI1283 | transcription elongation factor NusA | nusA | 2.067 | 0.000 | Other |
| HI1178 | arginine transporter permease subunit ArtQ | artQ | 2.065 | 0.000 | Other |
| HI1605 | hypothetical protein |  | 2.052 | 0.000 | Other |
| HI1284 | translation initiation factor IF-2 | infB | 2.043 | 0.000 | Other |
| HI0792 | 30S ribosomal protein S8 | rpsH | 2.041 | 0.000 | Ribos |
| HI0791 | 30S ribosomal protein S14 | rpsN | 2.029 | 0.000 | Ribos |
| HI0790 | 50S ribosomal protein L5 | rplE | 2.011 | 0.000 | Ribos |
| HI0613 | L-fuculokinase | fucK | -2.013 | 0.000 | CRP-N |
| HI1617 | aromatic amino acid aminotransferase | aspC | -2.027 | 0.000 | Other |
| HI0495m | acid phosphatase/phosphotransferase | aphA | -2.030 | 0.000 | Other |
| HI0144 | N-acetylmannosamine kinase |  | -2.032 | 0.000 | Other |
| HI0234 | pseudogene |  | -2.054 | 0.000 | Other |
| HI0287 | tryptophan-specific transport protein | mtr | -2.061 | 0.000 | Trp |
| HI0365 | ribosomal RNA large subunit methyltransferase N |  | -2.068 | 0.000 | CRP-S |
| HI0050m | integral membrane protein transporter |  | -2.074 | 0.000 | CRP-N |
| HI0487 | hypothetical protein |  | -2.139 | 0.000 | Other |
| HI0857.1 | 6S RNA | ssrS | -2.149 | 0.000 | Other |
| HI0612 | fucose operon protein | fucU | -2.152 | 0.000 | CRP-N |
| HI0834 | fumarate reductase iron-sulfur subunit | frdB | -2.155 | 0.000 | CRP-N |
| HI0501 | D-ribose pyranase | rbsD | -2.157 | 0.000 | Other |
| HI1434.1 | cold shock-like protein | cspD | -2.164 | 0.000 | CRP-N |
| HI1218 | L-lactate permease | lctP | -2.173 | 0.000 | CRP-N |
| HI0833 | fumarate reductase subunit C | frdC | -2.187 | 0.000 | CRP-N |
| HI0611 | L-fuculose phosphate aldolase | fucA | -2.225 | 0.000 | CRP-N |
| HI0399 | cyclic 3',5'-adenosine monophosphate phosphodiesterase | icc | -2.234 | 0.000 | CRP-N |
| HI0832 | fumarate reductase subunit D | frdD | -2.238 | 0.000 | CRP-N |
| HI1029 | hypothetical protein |  | -2.254 | 0.000 | CRP-N |
| HI0752 | phosphoribosylformylglycinamidine synthase | purL | -2.270 | 0.000 | Pur |
| HI1207 | amidophosphoribosyltransferase | purF | -2.305 | 0.000 | Pur |
| HI1730 | hypothetical protein |  | -2.310 | 0.000 | Other |
| HI0835 | fumarate reductase flavoprotein subunit | frdA | -2.317 | 0.000 | CRP-N |
| HI1661 | 2-oxoglutarate dehydrogenase E2 component dihydrolipoamide succinyltransferase | sucB | -2.320 | 0.000 | CRP-N |
| HI1729m | LamB/YcsF family protein |  | -2.322 | 0.000 | Other |
| HI1206 | colicin V production protein | cvpA | -2.340 | 0.000 | Pur |
| HI0140 | N-acetylglucosamine-6-phosphate deacetylase | nagA | -2.417 | 0.000 | CRP-N |
| HI0610 | L-fucose permease | fucP | -2.437 | 0.000 | CRP-N |
| HI1631 | competence gene of unknown function |  | -2.453 | 0.005 | CRP-S |
| HI1350 | cytidine deaminase | cdd | -2.531 | 0.000 | CRP-N |
| HI0143 | hypothetical protein |  | -2.538 | 0.000 | Other |
| HI0148.1 | pseudogene |  | -2.554 | 0.000 | Other |
| HI1645 | fructose-1,6-bisphosphatase | fbp | -2.562 | 0.000 | CRP-N |
| HI0366 | fimbrial biogenesis and twitching motility protein | pilF2 | -2.568 | 0.000 | CRP-S |
| HI1366 | acyl carrier protein phosphodiesterase | acpD | -2.672 | 0.000 | Other |
| HI1107 | Na+/H+ antiporter | nhaC | -2.690 | 0.000 | CRP-N |
| HI0804 | pseudogene |  | -2.708 | 0.000 | CRP-N |
| HI0035 | hypothetical protein |  | -2.747 | 0.000 | CRP-N |
| HI0147 | hypothetical protein |  | -2.750 | 0.000 | CRP-N |
| HI0831 | monofunctional biosynthetic peptidoglycan transglycosylase | mtgA | -2.776 | 0.000 | Other |
| HI0145 | N-acetylmannosamine-6-phosphate 2-epimerase |  | -2.795 | 0.000 | CRP-N |
| HI0257 | hypothetical protein |  | -2.849 | 0.000 | Other |
| HI1109 | D-xylose ABC transporter permease | xylH | -2.852 | 0.000 | CRP-N |
| HI1662 | 2-oxoglutarate dehydrogenase E1 component | sucA | -2.907 | 0.000 | CRP-N |
| HI0830 | Trp operon repressor | trpR | -2.912 | 0.000 | Trp |
| HI1398 | fumarate hydratase | fumC | -2.922 | 0.000 | Other |
| HI1124 | oligopeptide ABC transporter substrate-binding protein | oppA | -2.942 | 0.000 | CRP-N |
| HI0503 | ribose ABC transporter permease | rbsC | -2.958 | 0.000 | Other |
| HI0125 | hypothetical protein |  | -3.055 | 0.000 | Other |
| HI1028 | hypothetical protein |  | -3.095 | 0.000 | CRP-N |
| HI0141 | glucosamine-6-phosphate deaminase | nagB | -3.106 | 0.000 | CRP-N |
| HI0051 | hypothetical protein |  | -3.123 | 0.000 | CRP-N |
| HI1316 | sulfatase-like protein |  | -3.138 | 0.000 | CRP-N |
| HI1030 | hypothetical protein |  | -3.228 | 0.000 | CRP-N |
| HI1245 | malic enzyme |  | -3.266 | 0.000 | CRP-N |
| HI0434 | competence protein F | comF | -3.271 | 0.000 | CRP-S |
| HI0660 | competence toxin |  | -3.395 | 0.000 | CRP-S |
| HI1210 | malate dehydrogenase | mdh | -3.415 | 0.000 | CRP-N |
| HI0296 | type 4 prepilin-like protein specific leader peptidase | pilD | -3.421 | 0.000 | CRP-S |
| HI0690 | glycerol uptake facilitator protein | glpF | -3.424 | 0.000 | Other |
| HI0142 | N-acetylneuraminate lyase | nanA | -3.445 | 0.000 | Other |
| HI1110 | xylose transporter ATP-binding protein | xylG | -3.453 | 0.000 | CRP-N |
| HI0667 | fructose 1,6-bisphosphatase II | glpX | -3.454 | 0.000 | Other |
| HI1112 | xylose isomerase | xylA | -3.464 | 0.000 | CRP-N |
| HI0659 | competence antitoxin |  | -3.465 | 0.000 | CRP-S |
| HI1108 | pseudogene | patB | -3.482 | 0.000 | CRP-N |
| HI1183 | periplasmic ATP-dependent DNA ligase | ligA | -3.508 | 0.000 | CRP-S |
| HI1615 | phosphoribosylaminoimidazole carboxylase catalytic subunit | purE | -3.537 | 0.000 | Pur |
| HI1031 | 2,3-diketo-L-gulonate reductase |  | -3.588 | 0.000 | CRP-N |
| HI0815 | universal stress protein A | uspA | -3.588 | 0.000 | CRP-N |
| HI1390 | hydrogenase 2 accessory protein | hybG | -3.612 | 0.000 | Trp |
| HI0614 | L-fucose isomerase | fucI | -3.665 | 0.000 | CRP-N |
| HI0822 | galactose ABC transporter substrate-binding protein | mglB | -3.682 | 0.000 | CRP-N |
| HI0691 | glycerol kinase | glpK | -3.722 | 0.000 | Other |
| HI0809 | phosphoenolpyruvate carboxykinase | pckA | -3.727 | 0.000 | CRP-N |
| HI0435 | competence protein E | comE | -3.754 | 0.000 | CRP-S |
| HI1315 | hypothetical protein |  | -3.866 | 0.000 | CRP-N |
| HI0061 | recombination protein | rec2 | -3.897 | 0.000 | CRP-S |
| HI0745 | L-asparaginase II | ansB | -3.904 | 0.000 | CRP-N |
| HI1726 | phosphoribosylaminoimidazole-succinocarboxamide synthase | hemH | -3.923 | 0.000 | Pur |
| HI0131 | ferric ABC transporter protein | afuA | -3.990 | 0.000 | CRP-N |
| HI1360 | glycogen synthase | glgA | -4.008 | 0.000 | CRP-N |
| HI0941 | prepilin peptidase dependent protein C | comQ | -4.009 | 0.000 | CRP-S |
| HI1358 | glycogen operon protein | glgX | -4.104 | 0.000 | CRP-N |
| HI0888 | phosphoribosylamine--glycine ligase | purD | -4.114 | 0.000 | Pur |
| HI1388.1 | hypothetical protein |  | -4.116 | 0.000 | Trp |
| HI1430 | short chain dehydrogenase/reductase |  | -4.175 | 0.000 | Trp |
| HI1428 | phosphoribosylglycinamide formyltransferase | purN | -4.176 | 0.000 | Pur |
| HI1431 | tryptophan synthase subunit beta | trpB | -4.214 | 0.000 | Trp |
| HI1117 | competence protein | comM | -4.232 | 0.000 | CRP-S |
| HI0952 | DNA repair protein RadC | radC | -4.340 | 0.000 | CRP-S |
| HI0940 | function unknown | comP | -4.343 | 0.000 | CRP-S |
| HI1359 | glucose-1-phosphate adenylyltransferase | glgC | -4.346 | 0.000 | CRP-N |
| HI1356 | 4-alpha-glucanotransferase | malQ | -4.351 | 0.000 | CRP-N |
| HI0504 | D-ribose transporter subunit RbsB | rbsB | -4.351 | 0.000 | CRP-N |
| HI1111 | D-xylose transporter subunit XylF | xylF | -4.372 | 0.000 | CRP-N |
| HI0534 | aspartate ammonia-lyase | aspA | -4.423 | 0.000 | CRP-N |
| HI0146 | hypothetical protein |  | -4.498 | 0.000 | CRP-N |
| HI1389.1 | bifunctional indole-3-glycerol phosphate synthase/phosphoribosylanthranilate isomerase | trpC | -4.538 | 0.000 | Trp |
| HI1357 | glycogen branching protein | glgB | -4.557 | 0.000 | CRP-N |
| HI0053 | zinc-type alcohol dehydrogenase |  | -4.566 | 0.000 | CRP-N |
| HI0052 | hypothetical protein |  | -4.582 | 0.000 | CRP-N |
| HI1387 | anthranilate synthase component I | trpE | -4.668 | 0.000 | Trp |
| HI1389 | anthranilate phosphoribosyltransferase | trpD | -4.752 | 0.000 | Trp |
| HI0148 | N-acetylneuraminic acid mutarotase |  | -4.797 | 0.000 | CRP-N |
| HI1432 | tryptophan synthase subunit alpha | trpA | -4.810 | 0.000 | Trp |
| HI0939 | prepilin peptidase dependent protein B | comO | -4.821 | 0.000 | CRP-S |
| HI0608 | di- and tricarboxylate transporter |  | -4.838 | 0.000 | CRP-N |
| HI1388 | anthranilate synthase component II | trpG | -4.861 | 0.000 | Trp |
| HI0601 | DNA transformation protein | tfoX | -4.964 | 0.000 | CRP-N |
| HI1429 | phosphoribosylaminoimidazole synthetase | purM | -5.022 | 0.000 | Pur |
| HI0437 | competence protein C | comC | -5.253 | 0.000 | CRP-S |
| HI0436 | competence protein D | comD | -5.278 | 0.000 | CRP-S |
| HI0299 | prepilin peptidase-dependent protein D | pilA | -5.292 | 0.000 | CRP-S |
| HI0887 | bifunctional phosphoribosylaminoimidazolecarboxamide formyltransferase/IMP cyclohydrolase | purH | -5.363 | 0.000 | Pur |
| HI0938 | prepilin peptidase dependent protein A | comN | -5.402 | 0.000 | CRP-S |
| HI0438 | competence protein B | comB | -5.438 | 0.000 | CRP-S |
| HI0297 | type IV pilin secretion protein | pilC | -5.484 | 0.000 | CRP-S |
| HI1008 | competence protein ComEA | comE1 | -5.824 | 0.000 | CRP-S |
| HI0439 | competence protein A | comA | -6.034 | 0.000 | CRP-S |
| HI0298 | protein transport protein | pilB | -6.086 | 0.000 | CRP-S |
| HI0985 | DNA processing chain A | dprA | -6.226 | 0.000 | CRP-S |

**Supplementary Table E**

***H. influenzae* genes whose RNA-seq expression was significantly altered in ∆*toxA* relative to wildtype, to ∆*toxT* and to ∆∆*toxTA***

| **Gene ID** | **Gene name** | **Gene function** |
| --- | --- | --- |
| t=0 min |  |  |
| HI0655 | *aroE* | shikimate 5-dehydrogenase |
| HI0656.1 |  | hypothetical protein |
| HI0658 |  | ABC transporter ATP-binding protein |
|  |  |  |
| t=10 min |  |  |
| HI0231 | *deaD* | ATP-dependent RNA helicase |
| HI0655 | *aroE* | shikimate 5-dehydrogenase |
|  |  |  |
| t=30 min |  |  |
| HI0229 | *pnp* | polynucleotide phosphorylase/polyadenylase |
| HI0235 |  | hypothetical protein |
| HI0362 | *yfeA* | iron-chelated ABC transporter substrate-binding protein |
| HI0504 | *rbsB* | D-ribose transporter subunit RbsB |
| HI0595 | *arcC* | carbamate kinase |
| HI0655 | *aroE* | shikimate 5-dehydrogenase |
| HI1388 | *trpG* | anthranilate synthase component II |
| HI1633 | *purA* | adenylosuccinate synthetase |
|  |  |  |
| t=100 min |  |  |
| HI0125 |  | hypothetical protein |
| HI0146 |  | hypothetical protein |
| HI0148 |  | N-acetylneuraminic acid mutarotase |
| HI0153 | *dcuB* | pseudogene |
| HI0229 | *pnp* | polynucleotide phosphorylase/polyadenylase |
| HI0235 |  | hypothetical protein |
| HI0292 | *merP* | mercuric ion scavenger protein |
| HI0295 | *rho* | transcription termination factor Rho |
| HI0350 |  | permease |
| HI0361 | *yfeB* | iron (chelated) transporter ATP-binding protein |
| HI0362 | *yfeA* | iron-chelated ABC transporter substrate-binding protein |
| HI0478 | *atpC* | F0F1 ATP synthase subunit epsilon |
| HI0504 | *rbsB* | D-ribose transporter subunit RbsB |
| HI0514 | *rpoC* | DNA-directed RNA polymerase subunit beta' |
| HI0574 | *fkpA* | FKBP-type peptidyl-prolyl cis-trans isomerase |
| HI0595 | *arcC* | carbamate kinase |
| HI0596 | *arcB* | ornithine carbamoyltransferase |
| HI0654 | *tagI* | DNA-3-methyladenine glycosylase |
| HI0655 | *aroE* | shikimate 5-dehydrogenase |
| HI0656 |  | hypothetical protein |
| HI0656.1 |  | hypothetical protein |
| HI0658 |  | ABC transporter ATP-binding protein |
| HI0685 | *glpA* | sn-glycerol-3-phosphate dehydrogenase subunit A |
| HI0717 | *nusG* | transcription antitermination protein NusG |
| HI0761 | *mltC* | murein transglycosylase C |
| HI0811 | *argH* | argininosuccinate lyase |
| HI0814 | *alaS* | alanyl-tRNA synthetase |
| HI0846 | *por* | oxidoreductase |
| HI1144 | *lpxC* | UDP-3-O-[3-hydroxymyristoyl] N-acetylglucosamine deacetylase |
| HI1264 | *gyrA* | DNA gyrase subunit A |
| HI1336 | *folP-1* | dihydropteroate synthase |
| HI1345 | *potC* | spermidine/putrescine ABC transporter membrane protein |
| HI1354 | *glnS* | glutaminyl-tRNA synthetase |
| HI1464 | *folP-2* | dihydropteroate synthase |
| HI1525 |  | molybdate-binding periplasmic protein |
| HI1622 | *nikL* | nickel transport protein |
| HI1624 | *nikK* | nickel operon regulatory protein |

**SUPPLEMENTARY FIGURES:**

**Supplementary Figure A:** **Growth and competence phenotypes of *A. pleuropneumoniae* wildtype and *toxTA* deletion strains. A-1:** Transformation after transfer to competence-inducing conditions. MIV-induced cultures were incubated with novobiocin resistant (Nov^R^) chromosomal DNA, and transformation frequencies were calculated as Nov^R^ colonies per colony forming unit (CFU). Bars represent the means of at least three biological replicates, with error bars representing one standard deviation. A-2. **Growth in rich medium.** Blue: Wildtype, orange: ∆*toxA*, grey: ∆*toxT*, yellow: ∆*toxTA*. Each data point represents the average of 20 replicate wells.

**Supplementary Figure B. Development of competence by *H. influenzae* strains in MIV competence medium.**  Blue, wildtype (strain KW20); green, ∆*hfq* (strain RR3187); orange, ∆*toxA* (RR3158).

**Supplementary Figure C:**  **Bioscreen analysis of growth of *H. influenzae* wildtype and *toxTA* deletion strains in rich medium.** Blue: KW20, orange: ∆toxA, grey: ∆toxT, yellow: ∆toxTA. Each data point represents the average of 20 replicate wells.

**Supplementary Figure D. Growth of *H. influenzae* wildtype and ∆*toxA* strains after transfer to MIV competence medium.**  Blue, wildtype (strain KW20); orange, ∆*toxA* (RR3158).

**Supplementary Figure E:**  **Growth and MIV recovery of *H. influenzae* KW20 and ∆toxA** **measured by OD_600_.** Log-phase cells in sBHI were transferred to MIV at t=65 min; a portion of each MIV culture was diluted 10-fold into sBHI at t=170 min. The grey-shaded area indicates samples taken from MIV cultures. Blue: KW20, orange: ∆toxA. Dots are data from 2 independent experiments.

**Supplementary Figure F:** **Effect of cAMP on *H. influenzae* *toxTA* mutants.** Transformation was assayed 45 min after addition of cAMP (1 mM) to sBHI cultures. Error bars are standard deviations for two replicate cultures. * indicates values below the detection limit.

**Supplementary Figure G: Transformation frequencies of wildtype and ∆*toxT* cells growing in rich medium.** Solid symbols and black line: wildtype (KW20), open symbols and grey line: ∆*toxT*. Lines were fitted by eye. Diamonds and circles indicate data from independent experiments.

**Supplementary Figure H: Competence-induced expression of *H. influenzae* *toxA*.** Sample FPKM values (dots) and means (lines) for *toxT* (HI0660). Strains: wildtype: green; ∆*crp*: brown; ∆*sxy*: blue; ∆*toxA*: red; ∆*toxT*: grey. The values for the ∆*toxA* samples are underestimates because most of the gene has been deleted in this strain.

**Supplementary Figure I: Effect of hypercompetence mutations on expression of *H. influenzae* *toxA* and *toxT.*** Wildtype cells (KW20) and hypercompetent mutant cells (*murE749*, *rpoD-1* and *sxy-1*) were cultured in sBHI and sampled for RNA-seq analysis at OD_600_ densities of 0.02, 0.6 and 1.0. Upper panel, expression of *toxA* (HI0659); lower panel, expression of *toxT* (HI0660). Note that the Y-axis uses a log scale. Error bars indicate the standard errors of the replicate samples.

**Supplementary Figure J: Effects of ∆*toxTA* mutations on expression of competence regulator genes.** Panels: **A.** *Sxy* expression, **B.** *cya* expression, **C.** *crp* expression. Strains: orange: wildtype; blue: ∆*toxA*; yellow: ∆*toxT*; grey: ∆∆*toxTA*.

**Supplementary Figure K: Changes in expression levels of competence operons at t=100.** Strains: KW20 (green), ∆*toxA* (purple). All changes are relative to t=0. Black lines show standard errors.

**Supplementary Figure L:**  **RNA-seq coverage of *H. influenzae* *comNOPQ* after 30 min of competence induction.**  The green (wildtype cells) and purple (∆*toxA*) lines indicate mean coverage of the comNOPQ operon after 30 min in MIV, normalized by library size using DESeq2 [size factors] at each position; shaded areas indicate standard errors.

**References:**

1. Love M, Anders S, Huber W. Differential analysis of RNA-Seq data at the gene level using the DESeq2 package. Heidelberg: European Molecular Biology Laboratory (EMBL). 2013.
2. Redfield RJ, Cameron ADS, Qian Q, Hinds J, Ali TR, Kroll JS, *et al*. A novel CRP-dependent regulon controls expression of competence genes in *Haemophilus influenzae*. J Mol Biol. 2005;4(8):735-747.
3. Sinha S, Mell JC, Redfield R. The availability of purine nucleotides regulates natural competence by controlling translation of the competence activator Sxy. Mol Microbiol. 2013;88(6):1106-1119.
4. Iost I, Dreyfus M. DEAD-box RNA helicases in Escherichia coli. Nucleic Acids Res. 2006;34(15):4189–4197.
5. Garza-Sanchez F, Shaub RE, Janssen BD, Hayes CS. tmRNA regulates synthesis of the ArfA ribosome rescue factor. Mol Microbiol. 2011;80(5):1204-1219.
6. Taylor RG, Walker DC, McInnes RR. E. coli host strains significantly affect the quality of small scale plasmid DNA preparations used for sequencing. Nucleic Acids Res. 1993;21(7):1677-8.
7. Fleischmann RD, Adams MD, White O, Clayton RA, Kirkness EF, Kerlavage AR, *et al.* Whole-genome random sequencing and assembly of Haemophilus influenzae Rd. Science. 1995;269(5223):496-512.
8. Poje G, Redfield RJ. Transformation of *Haemophilus influenzae*. Methods Mol Med. 2003;71:57-70.
9. Sinha S, Mell JC, Redfield RJ. Seventeen Sxy-dependent cyclic AMP receptor protein site-regulated genes are needed for natural transformation in *Haemophilus influenzae*. J Bacteriol. 2012;194(19):5245-5254.
10. Chandler MS. The gene encoding cAMP receptor protein is required for competence development in *Haemophilus influenzae* Rd. P Natl Acad Sci USA. 1992;89(5):1626-30.
11. Williams PM, Bannister LA, Redfield RJ. The *Haemophilus influenzae* sxy-1 mutation is in a newly identified gene essential for competence. J Bacteriol. 1994;176(22):6789-6794.
12. Blackall PJ, Klaasen HL, van den Bosch H, Kuhnert P, Frey J. Proposal of a new serovar of *Actinobacillus pleuropneumoniae:* serovar 15. Vet Microbiol. 2002;84(1-2):47-52.
